# Study on Efficient Degradation of Waste PU Foam

**DOI:** 10.3390/polym15102359

**Published:** 2023-05-18

**Authors:** Xiaohua Gu, Xiaoyao Wang, Xinyu Guo, Siwen Liu, Chunhua Lou, Yan Liu

**Affiliations:** 1 School of Energy and Building Environment, Guilin University of Aerospace Technology, Guilin 541004, China; 2 School of Material Science and Engineering, Qiqihar University, Qiqihar 161006, China; 3 School of Materials Science and Engineering, Donghua University, Shanghai 200051, China; 4 College of Chemistry and Chemical Engineering, Qiqihar University, Qiqihar 161006, China; 5 College of Innovative Material & Energy, Hubei University, Wuhan 430062, China

**Keywords:** polyurethane foam, high-efficiency catalyst, recycling, glycolysis

## Abstract

In this paper, the high-efficiency degradation and alcoholysis recovery of waste polyurethane foam were realized using a combination of a high-efficiency alkali metal catalyst (CsOH) and two-component mixed alcoholysis agents (glycerol and butanediol) in different proportions, using recycled polyether polyol and one-step foaming to prepare regenerated thermosetting polyurethane hard foam. The foaming agent and catalyst were adjusted experimentally to prepare regenerated polyurethane foam, and a series of tests were conducted on the viscosity, GPC, hydroxyl value, infrared spectrum, foaming time, apparent density, compressive strength, and other properties of the degradation products of the regenerated thermosetting polyurethane rigid foam. The resulting data were analyzed, and the following conclusions were drawn: The optimal conditions of alcoholysis were obtained when the mass ratio of glycerol to butanediol was 3:2, the amount of cesium hydroxide was 0.08%, the reaction temperature was 170 °C, and the reaction time was 2.5 h. Regenerated polyurethane foam with an apparent density of 34.1 kg/m^3^ and a compressive strength of 0.301 MPa was prepared under these conditions. It had good thermal stability, complete sample pores, and a strong skeleton. At this time, these are the best reaction conditions for the alcoholysis of waste polyurethane foam, and the regenerated polyurethane foam meets various national standards.

## 1. Introduction

Since the invention of polyurethane foam by Otto Bayer in the early 1940s, its application has grown exponentially [1]. From bedding and upholstery materials to the electronics, automotive, and construction industries [2,3,4], the need to continuously improve the comfort and lifestyle of end consumers has been the driving force for this rapid expansion [5]. Polyurethane foam’s special comfort, insulation, resilience, and light weight are the driving factors for the development of the polyurethane foam market [6,7,8,9]. The global market demand for polyurethane products is expected to grow from about 15 million tons in 2020 to 20 million tons in 2025, with an annual growth rate of 7.5% between 2020 and 2025 [10,11,12,13,14,15]. However, at the same time, the production and use of a large number of waste products are one of the main sources of pollutants [16]; thus, dealing with a large amount of waste polyurethane has become a top priority. The physical treatment destroys the chemical structure of polyurethane to a certain extent [17,18,19], leading to a decline in the performance of the regenerated product. Biological treatment is time-consuming and inefficient, and alcoholysis is the most feasible method for chemical treatment [20,21,22]. The alcoholysis method, as the name implies, uses alcohols under the action of catalysts and other substances, under certain pressure and temperature conditions, to degrade waste polyurethane foam [23]. The essence is that the alcohol hydroxyl group and polyurethane chain segment on the ammonia ester bond alcohol exchange reaction, leading to the polyurethane molecular segment “cracking” into relatively small molecular weight alcohol substances (regenerated polyether polyol).

In this paper, alcoholysis was used to degrade waste polyurethane foam. A high-efficiency catalyst, i.e., strongly basic CsOH, was used to catalyze the degradation of waste PU. The degradation conditions of waste polyurethane were analyzed using experiments, and after changing the reaction conditions and the amounts of reagents added, a series of tests examining the viscosity, hydroxyl value, and infrared spectrum was carried out on the degradation products. The data were analyzed so as to obtain the optimal reaction conditions for the alcoholysis of waste polyurethane foam, which lays a certain theoretical basis for further exploration and research on the efficient degradation of waste polyurethane using catalysts.

## 2. Experimental Section

### 2.1. Raw Materials and Reagents

The reagents used in the experiment are shown in Table 1.

### 2.2. Instruments and Equipment

The main instruments required for this experiment are shown in Table 2.

## 3. Experimental Methods

### 3.1. Degradation Experiment of Waste Polyurethane Foam

First, the waste polyurethane foam was pulverized into powder using a pulverizer. Certain amounts of glycerol and butanol were added to a 1000 mL three-neck reactor, followed by different proportions of alkali metal catalysts (CsOH/KOH). After the catalyst was completely dissolved in the alcoholysis agent, the waste polyurethane foam powder was heated and stirred in the heating sleeve. After a period of time, the spent polyurethane foam gradually dissolved, and the resulting viscous liquid was the recycled polyol. The degradation products were poured out after cooling, and a series of tests and analyses were carried out on the hydroxyl value, viscosity, and infrared spectrum to determine the optimal reagent ratio.

### 3.2. Preparation of Regenerated Polyurethane Foam

The regenerated polyurethane foam was prepared with a one-step foaming method using the regenerated polyol and commercial polyether polyol 4110 as the main raw materials. Certain amounts of the prepared regenerated polyol (DM) and commercial polyether polyol 4110 were added into a disposable plastic cup, and then different amounts of dimethylsilicone oil (PDMS), foaming agent (CAS287-92-3), catalyst triethanolamine (TEA), and dibutyltin dilaurate (DBTDL) were added into the cup. Stirring was carried out using a cantilever mixer with a stirring rate of 1500 rpm and a stirring time of 30 s. Then, polyisocyanate (PAPI) was added to the mixture, and stirring was continued until foaming was observed. The produced recycled PU foam was placed in a cool and dry place for 48 h and then tested.

## 4. Testing and Characterization

### 4.1. Viscosity Test

The prepared degradation product was poured into a clean beaker. A rotary viscometer was then used to test the viscosity of the degradation product. The degradation product was completely under the probe during the test, and the viscosity data were recorded at 25 °C.

### 4.2. Hydroxyl Value Test

Appropriate degradation products were placed in a 10 mL conical flask, and the hydroxyl value was determined using the pyridine method according to the GB/T12008.3-2009 standard.

### 4.3. Fourier Transform Infrared Spectrum Analysis

GR-285 IR produced by Dalian Precision Scientific Instruments Co., Ltd. (Dalian, China). was used to analyze the structure of the foam samples, and the sampleswere formed by the KBr pressing method; the test wavelength range was 500–4000 cm^−1^.

### 4.4. Gel Permeation Chromatography Analysis

Gel permeation chromatography was used to test the molecular weight distribution of the degradation products. A certain amount of degraded regenerated polyols was added into dimethylformamide and mixed. Gel permeation chromatography was then used to measure the samples for testing and analysis, so as to determine the molecular weight of the degradation products and explore the relationship between the weight and properties of the degradation products.

### 4.5. Apparent Density Test

The apparent density of the regenerated polyurethane foam was tested according to the GB/T6343-2009 standard. After standing for 72 h, the regenerated polyurethane foam sample was cut into 50 mm × 50 mm × 50 mm sample blocks, and the sample blocks were weighed using an electronic balance. The density of the regenerated polyurethane foam was calculated using the formula ρ = m/V (ρ-Density, m-Mass, V-Volume). Each group of regenerated polyurethane foam was used to prepare five samples from which the average value was calculated.

### 4.6. Compression Strength Test

The compressive strength of the regenerated polyurethane foam was tested according to GB/T 8813-2008. The test instrument was an HKW 50 kn universal material testing machine, the sample size was 50 mm × 50 mm × 50 mm, and the displacement speed of the testing machine was 20 mm/min. Five measurements were made for each group of samples from which the average value was calculated.

### 4.7. Scanning Electron Microscope

The regenerated polyurethane foam of each group was sliced, and the samples were vacuumized and gold sprayed on the loading platform. The framework and pore structure in each regenerated polyurethane foam sample were observed with different multiples.

## 5. Results and Discussion

### 5.1. Discussion on the Degradation Mechanism of Waste Polyurethane

**Figure 1:**
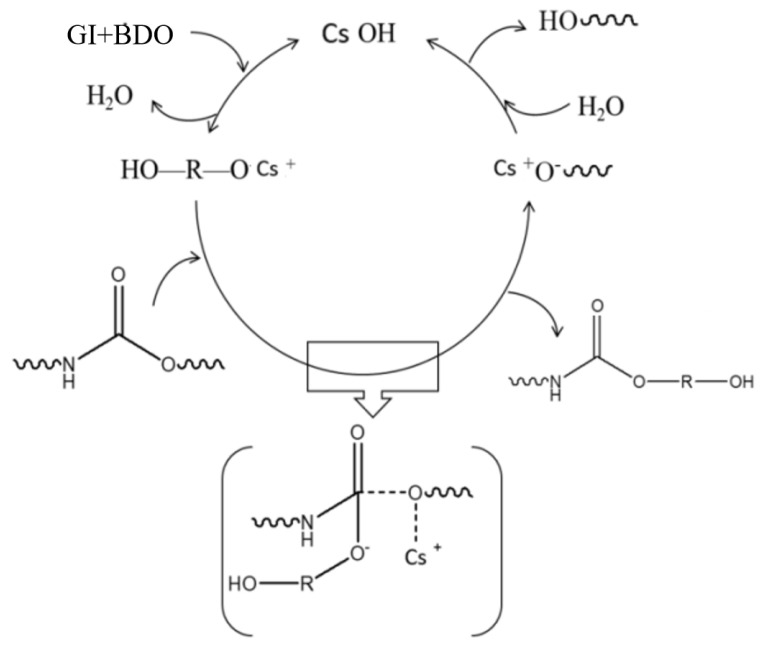
Degradation reaction of waste polyurethane.

The reaction mechanism of waste polyurethane degradation is shown in Figure 1.

### 5.2. Selecting the Proportions of Alcoholysis Agents

**Figure 2:**
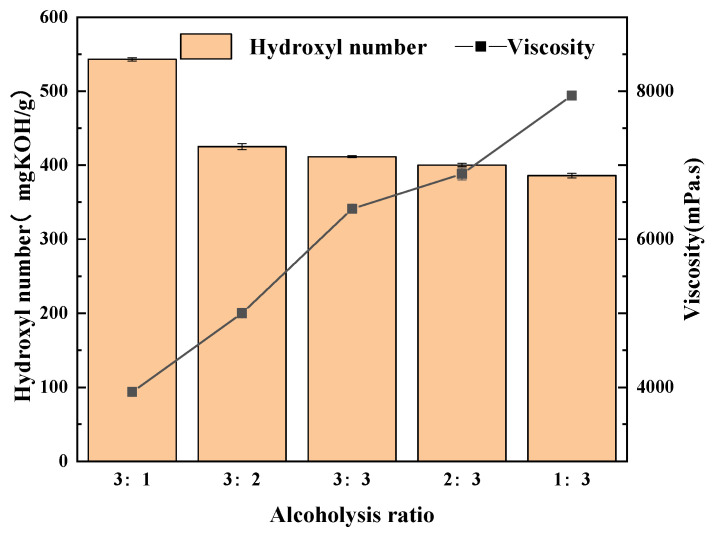
Hydroxyl value and viscosity of regenerated polyols with different alcohol ratios.

Using glycerol and butanediol as alcoholysis agents, the tested ratios of the two alcohols were 3:1, 3:2, 3:3, 2:3, and 1:3; the ratio of alcoholysis agent to waste PU foam was 1:1; and the amount of potassium hydroxide catalyst was 0.5%. The reaction was conducted at 170 °C for 2.5 h to explore the influence of the ratio of alcoholysis agents on the degradation products. The hydroxyl value and viscosity of the regenerated polyols prepared with different proportions of alcoholysis agents are shown in Figure 2.

As can be seen from Figure 2, with the decrease in the proportion of butanediol, the hydroxyl group value of the degraded and regenerated polyols decreased first significantly and then slightly with little change. The viscosity first decreased and then increased with the decrease in glycerin proportion. The hydroxyl number of the degradation products was affected by the degradation of polyols, glycerol, and butanediol as alcoholysis agents. When the glycerol/butanediol ratio was 3:1, the hydroxyl value of the degradation product was too high, indicating that the glycerol reaction was incomplete. However, the hydroxyl values of the subsequent groups were similar to those of commercially available polyether polyol 4110 indicating complete degradation. The more thorough the degradation, the lower the viscosity of the degraded product [24]. In summary, the best ratio of alcoholysis agents is 3:2 glycerol/butanediol to achieve the best reaction effect.

### 5.3. Choice of Foam and Alcoholysis Agent Ratio

**Figure 3:**
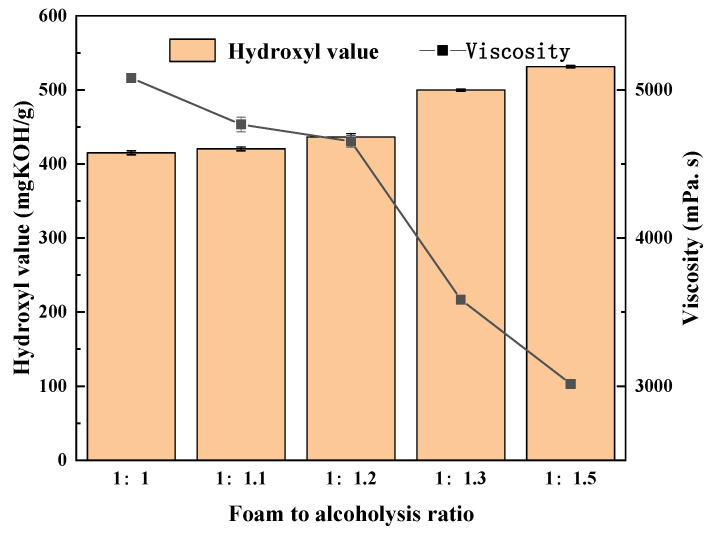
Hydroxyl value and viscosity of regenerated polyols with different foam-to-alcohol ratios.

Glycerol and butanediol in a mass ratio of 3:2 were used as alcoholysis agents, and 0.5% of total mass potassium hydroxide was added as a catalyst. The reaction temperature was set at 170 °C, and the reaction time was 2.5 h for the experiment. The mass ratio of waste PU foam to alcoholysis agents was used as the variable, and ratios of 1:1, 1:1.1, 1:1.2, 1:1.3, and 1:1.5 were used to carry out the experiment. The hydroxyl value and viscosity of the obtained polyols were then tested and analyzed. The result is shown in Figure 3.

As can be seen from Figure 3, the hydroxyl value of the degraded polyols increased with increasing amounts of alcoholysis agents. When the ratio between waste PU foam and alcoholysis agents was 1:1, 1:1.1, and 1:1.2, the hydroxyl value increased steadily and slightly to 445 mgKOH/g, and when the ratio between waste PU foam and alcoholysis agents reached 1:1.3, the hydroxyl value increased significantly to 483 mgKOH/g. This indicates the addition of excess alcoholysis agents at this time; after the degradation of polyols, some unreacted glycerol and butanediol remained, leading to the increase in the hydroxyl value. When the ratio of used PU foam to alcoholysis agents was 1:1, 1:1.1, and 1:1.2, the viscosity gradually dropped to 4678 mPa·s, indicating that the degradation was gradually completed. When the ratio of used PU foam to alcoholysis agents reached 1:3, the viscosity dropped sharply. At this time, the used PU foam was completely degraded and there was a certain amount of alcoholysis agents remaining, resulting in a significant decrease in viscosity [25]. In summary, when the ratio of waste PU foam to alcoholysis agents was 1:1.2, the degradation effect was the best, and there were no excess alcoholysis agents.

### 5.4. Research on the Amount of Cesium Hydroxide Catalyst

Catalysts play an important role in experiments on the degradation, recovery, and regeneration of PU foam using alcoholysis. The degradation effect determines the quality of the degraded polyols and their products [26,27], so it is necessary to test and analyze the amount of catalyst required and the performance of the products. Glycerol and butanediol in a mass ratio of 3:2 were selected as alcoholysis agents, the ratio of waste PU foam to alcoholysis agents was 1:1.2, the reaction temperature was set at 170 °C, and the reaction time was 2.5 h. Multiple experiments were carried out with different amounts of cesium hydroxide catalyst and compared with potassium hydroxide as the catalyst. Cesium hydroxide catalysts at 0.04%, 0.06%, 0.08%, 0.10%, and 0.12% of the alcoholysis agents and the total amount of waste PU foam and potassium hydroxide at 0.5% of the total amount were used as catalysts for degradation. The hydroxyl value and viscosity of the degraded polyols were tested. The results are shown in Table 3.

As can be seen from Table 3, with an increasing amount of cesium hydroxide catalyst added, the hydroxyl value and viscosity of the degraded regenerated polyols changed compared with those obtained using potassium hydroxide as the catalyst. Compared with that observed using potassium hydroxide at a mass ratio of 0.5%, the hydroxyl value of the regenerated polyol prepared with cesium hydroxide at various mass ratios was relatively small and similar to the hydroxyl value of commercial polyether polyol 4110, and it reached a higher value when the cesium hydroxide content ratio was 0.08%. The viscosity of the regenerated polyols first decreased and then increased with the addition of cesium hydroxide. The above data indicate that the addition of a cesium hydroxide catalyst can promote degradation when the amount is less than or equal to 0.08%. When the added amount is greater than 0.08%, the reaction is too violent, resulting in an incomplete reaction and decreased performance, but the degradation effect is higher than that of the 0.5% potassium hydroxide catalyst. In summary, the cesium hydroxide catalyst additive amount of 0.08% achieved the best effect.

### 5.5. Infrared Spectrum Analysis of Recovered Polyols

**Figure 4:**
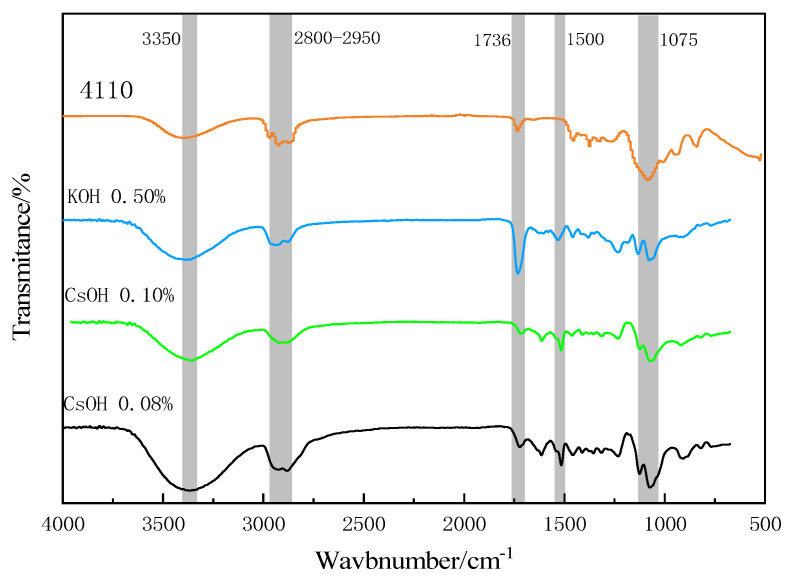
Infrared spectra showing the recycled polyol and polyether 4110.

The regenerated polyols and commercial polyether polyols 4110, which were degraded by adding different cesium hydroxide catalysts and potassium hydroxide catalysts, were examined using infrared spectrum analysis (Figure 4).

It can be seen from Figure 4 that the characteristic peaks in the regenerated polyols degraded with the cesium hydroxide catalyst and potassium hydroxide catalyst in different proportions were very similar to those of commercial polyether polyol 4110. There was a strong stretching vibration peak in the hydroxyl group at 3350 cm^−1^ [28] and a stretching vibration peak in the methyl group near 2900 cm^−1^ [29]. There was a characteristic absorption peak of the carbonyl group near 1737 cm^−1^ [30]. Unlike commercial 4110, the prepared regenerated polyol contained a benzene flooding peak near 1500 cm^−1^, which indicates that commercial polyether polyol does not contain a benzene ring [31]. There was an absorption peak near 1050 cm^−1^, which is the characteristic peak of the ether bond. These results indicate that the polyurethane bond in the waste polyurethane foam was broken and replaced with the alcohol hydroxyl group under the action of alcoholysis, and a regenerated polyol mixture containing ether bonds was generated. The regenerated polyol is similar in structure to the commercial polyether polyol 4110 and can be substituted for polyurethane foam.

### 5.6. Gel Permeation Chromatography Analysis

Table 4 shows the gel permeation chromatographic test results for the commercial polyether polyols 4110 and regenerated polyols produced using catalyst degradation of polyurethane waste foam.

It can be seen from Table 4 that the number average molecular weight of the regenerated polyols obtained using different catalysts and addition amounts ranged from 2511 to 3827. The more cesium hydroxide catalyst added, the smaller the molecular weight, and it was always smaller than the molecular weight of the regenerated polyols obtained with degradation using traditional potassium hydroxide as the catalyst. At the same time, with an increase in the cesium hydroxide catalyst amount, the distribution coefficient of the regenerated polyols gradually decreased to 1.168, which is close to that of polyether 4110. When the cesium hydroxide catalyst additive amount reached 0.08%, the change in the number average molecular weight and distribution coefficient of the regenerated polyols became small.

### 5.7. Effect of Foaming Catalyst and Additive Content on Regenerated Polyurethane Foam

#### 5.7.1. Effect of Foaming Agent Addition Amount on Regenerated Polyurethane Foam

A foaming agent is added in the preparation process for regenerated polyurethane foam. The action mechanism is that the foaming agent turns into gas when the reaction of polyol and polyisocyanate is heated, and the foam holes of the polyurethane foam are enlarged to achieve a foaming effect [32]. The optimum amount of foaming agent was determined by changing the amount of foaming agent. A disposable plastic cup was filled with 21.00 g polyether polyol 4110, 9.00 g recycled polyol, 0.6 g dimethyl silicone oil, 0.45 g triethanolamine, and 0.15 g dibutyl tin dilaurate. Then, foaming agents of different qualities were added (CAS287-92-3), and the mixture was stirred with a cantilever mixer for more than 30 s until evenly mixed. A quantity of 39.00 g polyisocyanate (PAPI) was added and stirred until foaming. The foaming time was measured, and the apparent density and compressive strength of the prepared regenerated polyurethane foam were tested.

(1) Foaming time: The time was recorded by adding polyisocyanate (PAPI) and stirring until the surface of the regenerated polyurethane foam hardened and no longer adhered. Each group of experiments was repeated five times from which the average value was calculated. The experimental results are shown in Table 5.

As can be seen from Table 5, with an increase in the amount of foaming agent added, the foaming time gradually accelerated. When the amount of foaming agent added increased from 4.50 g to 9.00 g, the foaming speed increased steadily, and when the amount of foaming agent added reached 10.50 g, the foaming speed increased significantly, indicating that the amount of foaming agent added has a significant impact on the foaming time. In addition, when the amount of foaming agent was 9.00 g, the foaming speed was moderate, and it was easy to control the reaction speed. When the amount of foaming agent was too much, the foam solidification time was too short, which is not suitable for practical applications.

**Figure 5:**
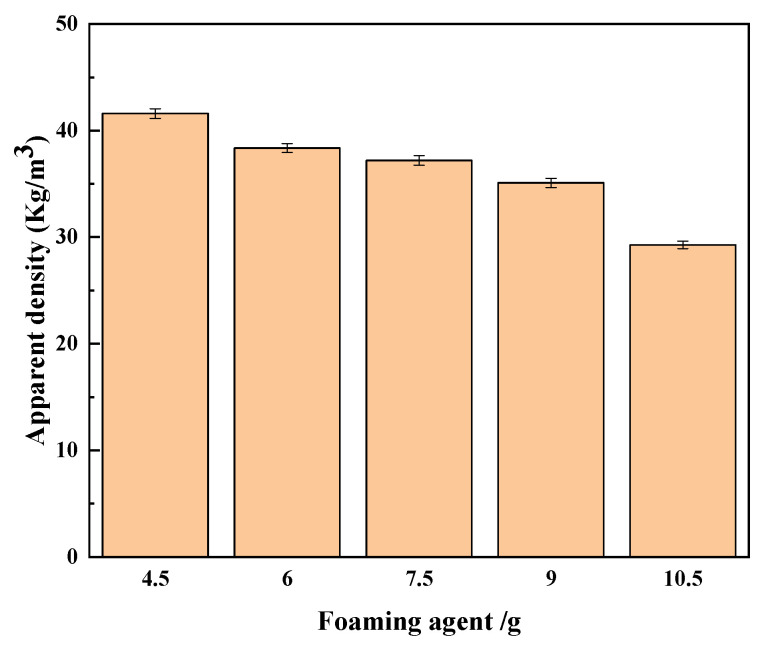
Apparent density of recycled polyurethane foam.

(2) Apparent density of regenerated polyurethane foam: After standing the prepared reclaimed polyurethane foam sample in a cool place for 72 h, the reclaimed polyurethane sample was cut into 50 mm × 50 mm × 50 mm sample blocks, and its apparent density was tested according to the GB/T6343-2009 standard. The sample blocks were weighed using an electronic balance, and the apparent density of the sample was then calculated using the formula ρ = m/V. The experiment was repeated five times for each group of samples from which the average value was calculated. The measurement results are shown in Figure 5.

As can be seen from Figure 5, the density of the regenerated polyurethane foam sample gradually decreased with the increase in the amount of foaming agent added. When the amount of foaming agent added exceeded 9.0 g, the density reduction increased substantially. This indicates that the optimal state was reached when the amount of foaming agent was 9.00 g, and the density of the regenerated polyurethane foam was 34.1 kg/m^3^; this is a lower density, but the foam also had a higher porosity and a better distribution of bubbles. When the amount of foaming agent was too much, the porosity of the regenerated polyurethane foam was too high, which affected the performance of the foam [33].

**Figure 6:**
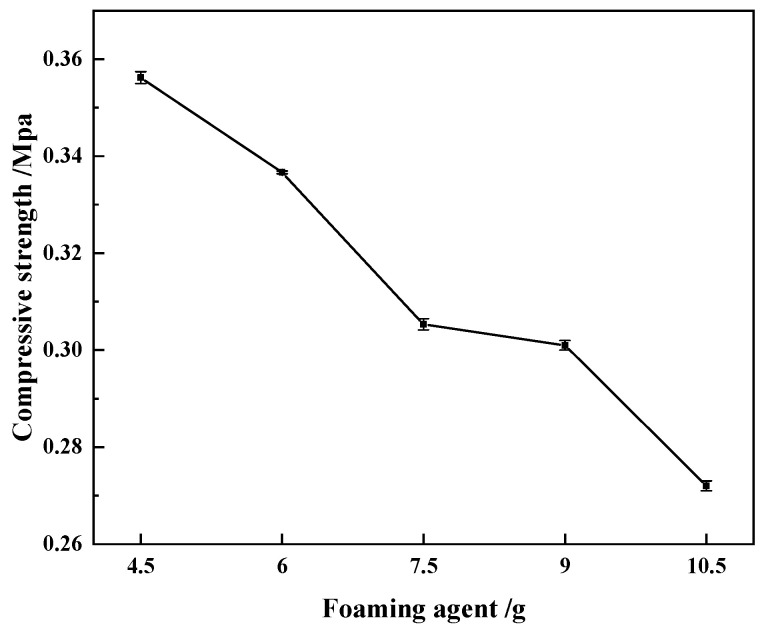
Compressive strength of recycled polyurethane foam.

(3) Compressive strength of regenerated polyurethane foam: The compressive strength of the regenerated polyurethane foam was tested according to GB/T 8813-2008. Samples with specifications of 50 mm × 50 mm × 50 mm were tested using a universal testing machine, and the displacement velocity of the testing machine was adjusted to 20 mm/min. Five measurements were made for each group of samples from which the average value was calculated. The test results are shown in Figure 6.

It can be seen from Figure 6 that the amount of foam added had an effect on the strength of the regenerated polyurethane foam. As the amount of foaming agent added increased, the compressive strength of the regenerated polyurethane foam gradually decreased, indicating that with an increase in the amount of foaming agent, the bubble holes gradually expanded, resulting in a slight decrease in the compressive strength. When the amount of foaming agent added reached 10.50 g, the compressive strength decreased significantly to 0.268 MPa. At this time, due to excessive foaming agent, the bubble holes were broken, and the compressive strength was greatly reduced. When the amount of foaming agent was 9.00 g, the compressive strength was 0.301 MPa. At this time, the regenerated polyurethane foam had a better foaming volume, but it also had a higher compressive strength, representing the best addition amount.

**Figure 7:**
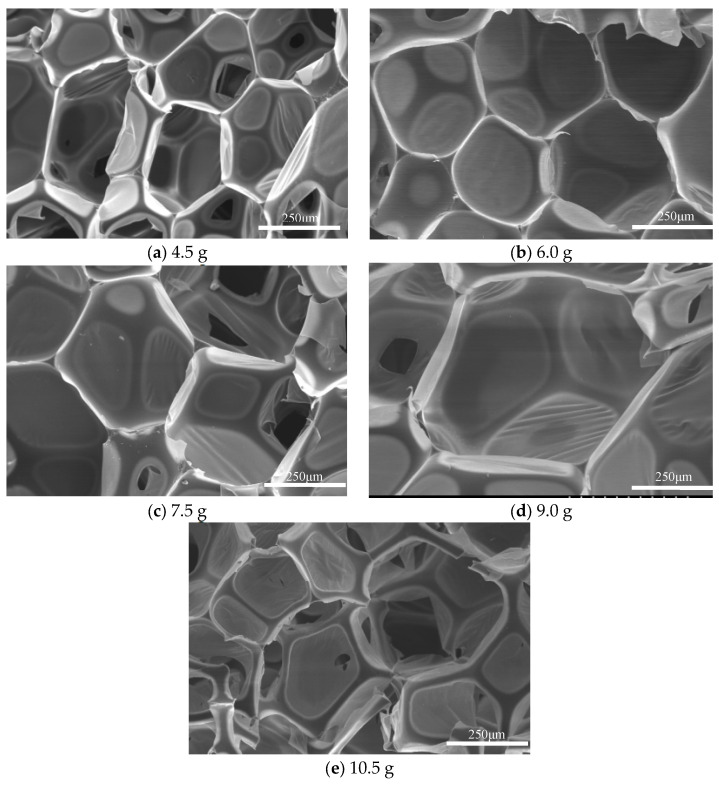
Scanning electron microscope images showing the polyurethane foams prepared with different amounts of foaming agent. (**a**–**e**): Respectively are foams prepared by adding different foaming doses.

(4) Scanning electron microscopy and analysis of regenerated polyurethane foam: The samples of regenerated polyurethane foams prepared with different amounts of foaming agent were sliced. Scanning electron microscopy was used to observe them, and clear areas were selected for shooting. The bubble holes in each sample are shown in Figure 7.

As can be seen from Figure 7, the foam holes in the regenerated polyurethane foam prepared with different amounts of foaming agent were different, and the volume of the foam holes gradually increased with an increasing amount of foaming agent [34]. When the amount of 4.50 g was added, the holes were small and shriveled, and their shape was irregular. When the amount of foaming agent reached 10.50 g, the bubble holes in the regenerated polyurethane foam reached their maximum size and breakage was observed. When the dosage of foaming agent was 9.00 g, the bubble holes were full and there was no breakage; the optimal dosage was reached at this time.

The results show that the foaming agent has significant effects on the foaming time, apparent density, and compressive strength of regenerated polyurethane foam. During the preparation of regenerated polyurethane foam, the foaming agent does not participate in the reaction, but when the reaction of polyol and polyisocyanate is heated, the foaming agent turns into gas, causing bubble hole expansion. With an increasing amount of foaming agent, the foaming time of the regenerated polyurethane foam gradually decreases, and the apparent density and compressive strength also decrease. However, when excess foaming agent is added, the bubble expansion will be too large, which damages the bubble holes by breaking their walls and results in a substantial reduction in strength. If the foaming reaction time is too fast, it is not suitable for mass production due to the lack of control over the molding rate. The addition of too much foaming agent also increases the production cost, so the appropriate addition of foaming agent can save resources on the basis of the best performance. In summary, the optimal addition level was 9.00 g.

#### 5.7.2. Effect of Catalyst Addition Amount on Regenerated Polyurethane Foam

During the preparation of regenerated polyurethane foam, a certain amount of catalyst is usually added to control the reaction rate of each raw material in the reaction; the most commonly used catalysts are tertiary amines and organotin [35]. Catalysts do not participate in the reaction during the preparation of regenerated polyurethane foam, they only accelerate or reduce the reaction rate between polyols and isocyanates. Usually, tertiary amine catalysts promote the foaming reaction, and organotin catalysts promote the gel reaction. The synergistic effect of the two catalysts can control the reaction rate within a proper range. In this experiment, triethanolamine and dibutyltin dilaurate were selected as catalysts. By adjusting the amounts of the two catalysts, the proportion of catalysts and the experimental reaction rate reached an optimal state of balance, so as to achieve the best performance of the recycled polyurethane foam.

A disposable plastic cup was filled with 21.00 g polyether polyol 4110, 9.00 g recycled polyol, 0.6 g dimethyl silicone oil, and 9.00 g foaming agent, and then different amounts of triethanolamine and dibutyl tin dilaurate were added. A jib mixer was used to stir the mixture for 30 s or more until evenly mixed. A quantity of 39.00 g polyisocyanate (PAPI) was added and stirred until foaming. After cooling and standing for 72 h, the prepared regenerated polyurethane foam was sliced, and its bubble holes were observed with a scanning electron microscope.

The amount of catalyst added, the size of regenerated polyurethane foam, and descriptions for the corresponding SEM images are shown in Table 6.

**Figure 8:**
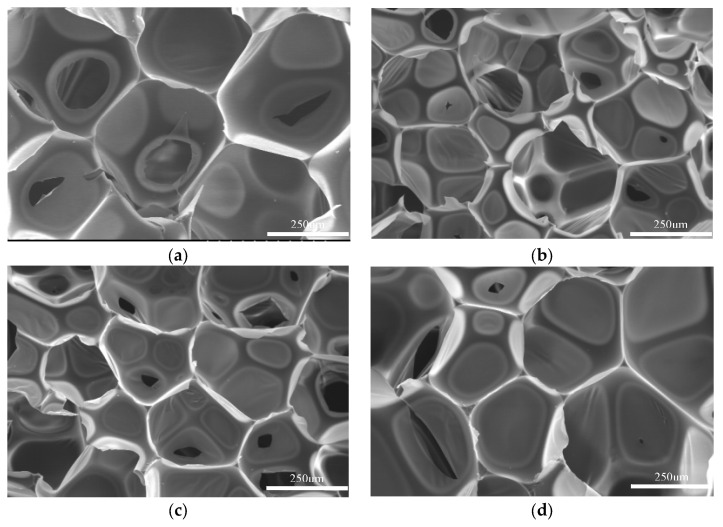

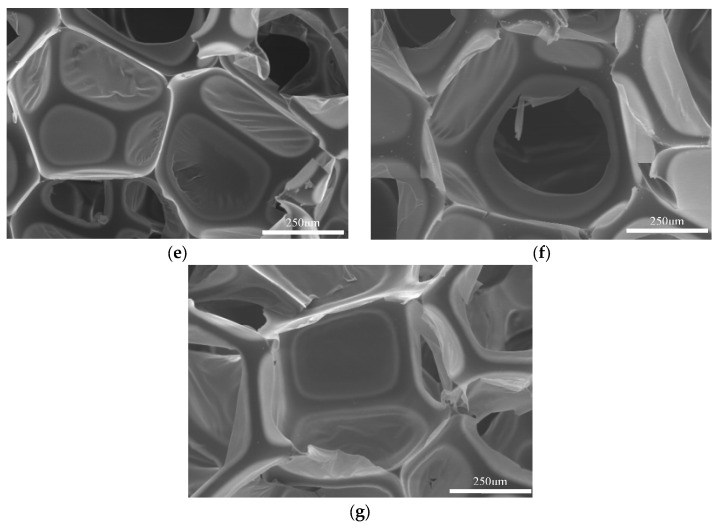
Scanning electron microscope images showing the recycled polyurethane foams prepared with different catalyst additions. (**a**–**g**) Details are described in Table 6.

As can be seen from Figure 8 and Table 6, the foam holes in regenerated polyurethane foams prepared with different catalyst addition amounts were slightly different. When the amount of both triethanolamine and dibutyltin dilaurate was 0.15 g in the sample (a), the volume of the prepared regenerated polyurethane foam was slightly smaller, and a collapse phenomenon occurred after standing. Scanning electron microscopy (a) showed that the pores were small and not full. The reason for this is that the added amount of dibutyltin dilaurate was slightly more, so the foaming reaction rate during the reaction was slightly less than the gel reaction rate. Thus, the foaming agent could not completely turn into gas and disperse and the bubble holes could not fully expand, which finally led to the collapse of the foam. Samples (b)–(d) were smaller in volume than sample (a) and also presented a collapse phenomenon. The scanning electron microscope images were also similar to that for sample (a). The pores were not full and were small, indicating that the addition of dibutyltin dilaurate in this component was excessive, the gel reaction was too fast, and the foaming was not complete. Sample (e) presented a foam shape of complete and moderate volume with no collapse after standing. Compared with other images, SEM image (e) showed a fuller bubble shape and stronger skeleton, and the effect was good. At this time, the addition ratio of triethanolamine and dibutyltin dilaurate was moderate, and the foaming reaction and gel reaction reached a balance. The volumes of samples (f) and (g) were larger than those of the previous groups, and they were more brittle and broken after pressing. In SEM (f) and (g), the bubble holes were very uneven, and some of the bubble holes were very large and even broken. The skeleton was thin, and a fracture phenomenon was observed. At this time, the amount of catalyst triethanolamine was excessive, the foaming reaction was too fast, and a large amount of foaming agent gas volatilization led to rapid enlargement, or even rupture, of the bubble holes.

According to the above experiments, the reaction reached the best state when the addition amount of triethanolamine was 0.30 g and the addition amount of dibutyltin dilaurate was 0.15 g. The prepared regenerated polyurethane foam had complete pores and a robust skeleton, indicating that the foaming reaction and gel reaction reached a balance. Under these conditions, the prepared regenerated polyurethane foam performed well.

## 6. Conclusions

In this paper, a high-efficiency alkali metal catalyst (CsOH) and two kinds and proportions of alcoholysis agents (glycerol and butanediol) were successfully used to efficiently degrade and recover waste polyurethane foam. Using a comprehensive analysis of the conditions in waste polyurethane degradation, recycled polyether polyol was used to prepare regenerated thermosetting polyurethane hard foam with one-step foaming. Optimal reaction conditions for the alcoholysis of waste polyurethane foam were obtained, and high-performance regenerated polyurethane foam was prepared. The conclusions are as follows:

(1) The optimal conditions of alcoholysis were obtained when the mass ratio of glycerol to butanediol was 3:2, the mass ratio of the used polyurethane foam to alcoholysis agents was 1:1.2, the amount of cesium hydroxide catalyst was 0.08%, the reaction temperature was 170 °C, and the reaction time was 2.5 h. Under these conditions, waste polyurethane was successfully degraded.
(2) When the amount of regenerated polyol was 9 g and the amount of polyether polyol 4110 was 21 g, adding 9.0 g of foaming agent, 0.30 g of triethanolamine catalyst, and 0.15 g of dibutyltin dilaurate produced regenerated polyurethane foam with a density of 34.1 kg/m^3^ and compression strength of 0.30 mpa. The regenerated polyurethane foam meets national standards, the sample bubble holes were complete, the skeleton was strong, and the best reaction conditions were achieved.

## Figures and Tables

**Table 1:** The main reagents of the experiment.

Reagent Name	Purity	Factory of Production
Waste polyurethane rigid foam	Industrial waste	Shanghai Hecheng Polymer Material Co., Ltd. (Shanghai, China)
Glycerine	AR	Tianjin Chemical Reagent Factory 1 (Tianjim, China)
Butane-1,4-diol (BDO)	AR	Tianjin Kaitong Chemical Reagent Co., Ltd. (Tianjin, China)
CsOH	AR	Shanghai Aladdin Biochemical Technology Co., Ltd. (Shanghai, China)
KOH	AR	Shanghai Aladdin Biochemical Technology Co., Ltd. (Shanghai, China)
Silicone oil stabilizer	CP	Guangzhou Feirui Chemical Co., Ltd. (Guangzhou, China)
TEA	AR	Shanghai Demao Chemical Co., Ltd. (Shanghai, China)
Dibutyltin dilaurate	AR	Shanghai Jieer Technology Co., Ltd. (Shanghai, China)
Foaming agent	CP	Shenzhen Huachang Chemical Co., Ltd. (Shenzhen, China)
Polyether 4110	CP	Shandong Lianhaoyao New Material Co., Ltd. (Shandong, China)
Polyaryl polymethylene isocyanate (PAPI)	CP	Wuhan Fude Chemical Co., Ltd. (Wuhan, China)

AR—analytical pure, CP—industrial grade.

**Table 2:** Experimental equipment used in this study.

Name of Instrument	Model	Factory of Production
Electronic analytical balance	JA3003C	Sartorius Scientific Instruments Co., Ltd. (Beijing China)
Cantilever constant-speed power electric mixer	TJ-1200W	Changzhou Huaao Instrument Manufacturing Co., Ltd. (Changzhou China)
Spherical reactor (1 L)	ZNHW-200	Shanghai Leighton Industrial Co., Ltd. (Shanghai, China)
Digital blast drying oven	WX881	Wujiang Weixin Electric Heating Equipment Co., Ltd. (Wujiang, China)
Digital viscometer	NDJ-5	Shanghai Pingxuan Scientific Instrument Co., Ltd. (Shanghai, China)
Disposable plastic cup	350 mL	Top Daily Chemicals Co., Ltd. (Yangzhou, China)
Constant-temperature heating sleeve	FDSG-420	Wuxi Huachang Chemical Co., Ltd. (Wuxi, China)

**Table 3:** Hydroxyl value and viscosity of regenerated polyols prepared using different catalysts and additives.

Additive Amount (%)	Hydroxyl Number/(mgKOH/g)	Viscosity (mPa·s)
0.04 (CsOH)	429	4498
0.06 (CsOH)	438	4254
0.08 (CsOH)	445	3834
0.10 (CsOH)	441	4432
0.12 (CsOH)	436	5139
0.50 (KOH)	437	4678

**Table 4:** The quantitative molecular weight (Mn) and molecular weight distribution coefficient (PDI) of regenerated polyols prepared using different catalysts and dosages.

Additive Amount/(%)	Mn	PDI
0.50 (KOH)	3827	1.466
0.04 (CsOH)	3794	1.401
0.06 (CsOH)	3526	1.324
0.08 (CsOH)	2804	1.267
0.10 (CsOH)	2683	1.269
0.12 (CsOH)	2511	1.274
Polyether 4110	1105	1.168

**Table 5:** Effect of the catalyst dosage ratio on the polyurethane foam structure.

Number	Foaming Agent/(g)	Cream Time/(s)	Tack-Free Time/(s)
1	4.50	3	58
2	6.00	2	53
3	7.50	3	50
4	9.00	3	48
5	10.50	2	39

**Table 6:** The effect of the catalyst dosage ratio on the sponge structure.

Sample	TEA/(g)	DBTDL/(g)	Morphology
a	0.15	0.15	Slightly smaller, cavity collapsed
b	0.15	0.30	Smaller, cavity collapse
c	0.15	0.45	Smaller, cavity collapse
d	0.30	0.45	Slightly smaller, cavity collapsed
e	0.30	0.15	Moderate, no hole collapse
f	0.45	0.30	Large, crispy skin
g	0.45	0.15	Large, crispy skin

## Data Availability

This study did not report any data.
